# Development and preliminary validation of Cancer-related Psychological Flexibility Questionnaire

**DOI:** 10.3389/fpsyg.2023.1052726

**Published:** 2023-03-02

**Authors:** Mei-jun Ou, Xiang-hua Xu, Hong Chen, Fu-rong Chen, Shuai Shen

**Affiliations:** ^1^Head and Neck Surgery Department, Hunan Cancer Hospital/The Affiliated Cancer Hospital of Xiangya School of Medicine, Central South University, Changsha, China; ^2^Health Service Center, Hunan Cancer Hospital/The Affiliated Cancer Hospital of Xiangya School of Medicine, Central South University, Changsha, China; ^3^School of Nursing, University of South China, Hengyang, China; ^4^Department of General Surgery, Changsha Hospital of Hunan Normal University/The Fourth Hospital of Changsha, Changsha, China

**Keywords:** psychological flexibility, cancer, questionnaire, cancer acceptance, cancer avoidance, activity engagement, valued action

## Abstract

The Cancer-related Psychological Flexibility Questionnaire (CPFQ) was developed and validated for assessing cancer patients’ psychological flexibility, including attitudes and behavior toward cancer. In a systematic process, the CPFQ identified four factors through principal component analysis and confirmatory factor analysis: Cancer Acceptance, Cancer Avoidance, Activity Engagement, and Valued Action. The results of this study reveal that the CPFQ has a clear factor structure and good psychometric properties. The specific nature of cancer and the need for a specific measure of cancer patient psychological flexibility make this questionnaire valuable for research on psychological flexibility in cancer patients.

## Introduction

Cancer is a leading cause of death and a worldwide public health issue. According to the GLOBOCAN 2020 estimates of cancer incidence and mortality worldwide in 185 countries, ~19.3 million new cancer cases and 10.0 million deaths occurred in 2020. Moreover, an estimated 28.4 million cases are expected in 2040 (Sung et al., 2021). Cancer is a chronic and life-threatening illness, and most cancer patients must undergo comprehensive anticancer treatment, including surgery, radiotherapy, and chemotherapy. Existing evidence showed that cancer patients often endure treatment-related toxicities and permanent functional impairment, which lead to multiple symptoms (Neufeld et al., 2017; Cuthbert et al., 2020; Gravier et al., 2020; Lage et al., 2020; Raphael et al., 2020). Cuthbert et al. reported that 60% and 80% of patients with a cancer diagnosis suffered from anxiety and low well-being, respectively (Cuthbert et al., 2020). Almost half of cancer survivors (43.6%) experienced a fear of relapse, and 32.1% had a severe/pathological fear of relapse in Singapore (Mahendran et al., 2021). A negative emotional state could significantly reduce the quality of life of cancer patients (Liu et al., 2021; Phoosuwan and Lundberg, 2022).

Many cancer patients adopt negative coping styles such as avoidance and pessimism when confronted with treatment-related toxicities, impaired functions, and distorted body image (Zhang et al., 2020). They might struggle to eliminate or fight cancer-related discomfort symptoms, including pain, fatigue, nausea, vomiting, dyspnea, and edema. Some live in this self-conceptualization and think they are useless and cumbersome. They experience a diminished sense of self-worth and show avoidance and withdrawal tendencies, such as avoiding discussing disease-related issues and reducing daily activities and social interactions. Coping styles of cancer survivors plays a predictive role in psychological symptoms, psychological well-being, and quality of life. The patients with an avoidance coping style showed higher cancer distress, anxiety, depression, and lower quality of life (Cheng et al., 2019). The better we understand the mechanism that underlies cancer patients’ avoidance coping style, the better we can reduce their psychological symptoms and improve their quality of life.

Psychological flexibility (PF), a new concept in clinical psychology, is defined as the ability to stay in contact with the present moment and pursue behavioral goals based on personal values and situational contexts, despite the presence of distress (Kashdan et al., 2020; Cherry et al., 2021). Psychological flexibility is a core construct of the Hexaflex model of acceptance and commitment therapy (ACT), one of the third wave of cognitive behavioral therapy (Hulbert-Williams et al., 2015). Acceptance and commitment therapy is based on Hexaflex model which is composed of six core components: acceptance, cognitive defusion, self as context, being present, values, and committed action. Acceptance and commitment therapy aims to improve the coping style and diminish the impact of adverse stressor events by deconstructing the individual experience in the context of personal values, enabling acceptance of both positive and negative responses (Hulbert-Williams et al., 2015), producing adaptive behavior change by enhancing PF (Hayes et al., 2019; Hofmann and Hayes, 2019). Many studies found that PF is associated with adaptive personality traits, including higher conscientiousness, openness to experiences, and lower negative emotionality, which may be the primary therapeutic mechanism of ACT (Hayes et al., 2006; Bryan et al., 2015). Previous studies indicated that a higher PF predicted lower anxiety, depression, and aversive emotional states in patients with breast cancer (Berrocal Montiel et al., 2016). A higher PF also resulted in a higher meaning in life in patients with thyroid cancer (Lv et al., 2021). In patients with prostate cancer, PF significantly predicted psychological distress and quality of life and moderated the relationship between the fear of recurrence and psychological distress (Sevier-Guy et al., 2021). Lucas et al. reported that PF was important for mental health and had a direct, positive effect on life satisfaction among community residents (Lucas and Moore, 2020). In conclusion, PF is a common protective factor across different contexts and populations. Therefore, quantitative assessment of PF for cancer patients can predict their psychological status and quality of life and evaluate the effect of ACT.

Psychological flexibility currently has a wide range of measurement tools. The most popular general measure of PF was AAQ-II, a version of the Acceptance and Action Questionnaire (AAQ; Cherry et al., 2021). AAQ-II has been widely used to measure PF in different contexts and populations, such as cancer patients (Lv et al., 2021) and community residents (Pyszkowska and Ronnlund, 2021). However, AAQ-II measures experiential avoidance (EA), which is an unwillingness to face unwanted experiences and acting to avoid them, and fails to capture core elements of PF (Kashdan et al., 2020). Experiential avoidance measured by AAQ-II is only a component of PF and cannot fully represent PF. In addition, there are many specific assessment tools for PF adapted from AAQ, such as the Acceptance and Action Diabetes Questionnaire (Gregg et al., 2007), the Chronic Pain Acceptance Questionnaire (CPAQ; Fish et al., 2010), and the Psychological Flexibility in Epilepsy Questionnaire (PFEQ; Burket et al., 2021), which are used to measure the PF of patients with diabetes mellitus, chronic pain, and epilepsy, respectively. However, there is no specialized assessment tool for PF in cancer patients.

Cancer is a chronic disease with long-term complex treatment and high physical and psychological burdens. Cancer is a life-threatening disease with high incidence, destruction of integrity, and high recurrence risk. Cancer patients are at risk for several comorbid psychological problems, such as anxiety, depression, and fear (Cuthbert et al., 2020; Lage et al., 2020; Raphael et al., 2020). Moreover, patients with cancer tend to confuse negative emotions and thoughts with objective facts, and immerse themselves in negative automatic thoughts, which aggravate negative emotions and form a vicious circle. Therefore, the PF of cancer patients may differ from that of patients with other non-cancer diseases owing to the characteristics of the tumor. The measure of PF in cancer patients is helpful in understanding their psychological process and coping style so that psychological interventions can be implemented to enhance PF, decrease psychosocial distress, and pursue a more meaningful and healthy life. In addition, it might reduce the specificity and sensitivity if general psychological flexibility assessment tools were used to measure PF of cancer patients. Hence, developing a self-reporting tool that specifically addresses PF in relation to cancer for both research and clinical purposes is necessary.

The present study aimed to (1) develop a tool to measure PF in cancer patients and identify its latent structure [Study 1], and (2) confirm the structure, and explore the validity of the questionnaire by using the Meaning in Life Questionnaire, the Templer’s Death Anxiety Scale, and the Acceptance and Action Questionnaire II [Study 2].

## Study 1: Questionnaire construction and development

### Materials and methods

#### Participants

Participants were recruited using a convenience sampling method from a tertiary cancer hospital in Hunan Province, China. Patients were included if they: (a) were aged over 18 years old, (b) had a diagnosis of cancer and awareness of it; (c) had normal cognitive function and were able to read and write; (d) could complete the survey; and (e) were willing to participate and provide informed consent. A total of 250 questionnaires were distributed. Finally, 231 patients completed the survey, with a valid response rate of 92.4%. Of all the patients, 115 were men, aged 19–89 years, with an average age of 56.4 ± 11.3 years. Most of them were married (90%) (*n* = 208), 4.3% (*n* = 10) were single, and 5.6% (*n* = 13) were divorced or widowed. As for educational background, 27.7% (*n* = 64) completed primary school or below, 42.8% (*n* = 99) junior high school, 14.3% (*n* = 33) senior high school, and 15.2% (*n* = 35) college and above. Regarding cancer stages, 4.3% (*n* = 10) of the patients had stage I, 24.2% (*n* = 56) stage II, 39.0% (*n* = 99) stage III, 15.6% (*n* = 36) stage IV, and 16.9% (*n* = 39) were unreported.

#### Item generation of the pilot Cancer-related Psychological Flexibility Questionnaire

There were four steps to generate the items of the pilot Cancer-related Psychological Flexibility Questionnaire. The four steps are item generation, scoring methodology, expert consultation, and pilot test, described as follows:

#### Item generation

The generation of items was based mainly on the following principles. (1) It came from the analysis of the Hexaflex theoretical framework and a large number of literature reviews. According to the model, PF included two processes, which consist of six core components: mindfulness and acceptance processes (acceptance, cognitive defusion, and self as context) and commitment and behavior change processes (being present, values, and committed action). (2) It followed items of other measurement tools of psychological flexibility, such as the CPAQ (Fish et al., 2010) and the Multidimensional Psychological Flexibility Inventory (MPFI; Rolffs et al., 2018). (3) Semi-structured interviews with open-ended questions were conducted with a representative sample of cancer patients to understand their feelings and responses after a cancer diagnosis. The interview outline revised by experts was as follows: ① Please describe your experience or feeling about cancer; ② What influences or changes have tumors brought to your life, including daily life, work, social interaction, family relations, etc.?; ③ What do you do in the face of cancer?; ④What are your main concerns?; and ⑤ What are your plans for the future?. An experienced interviewer conducted one-to-one interviews in an independent and quiet room. The entire interview process was recorded. The interviewer transcribed and analyzed the interview results on the day of the interview and stopped the interview after sufficient information was gathered. Finally, 18 cancer patients were interviewed. Four themes were extracted: negative emotions (distress, shame, frustration, anxiety, and self-blame), avoidance coping (avoid disease, social isolation, workplace alienation, and meaningless life), positive coping (accept reality, cooperate with treatment, and cherish life), and future plan (adjust lifestyle, assume roles, and go with the flow).

If an item reflected one of the six core components mentioned above, it was included in the potential items pool. Following these guidelines, 32 potential items were created to reflect the construct of PF. After study group discussion, some similar items were deleted or merged, and 14 items were retained.

#### Scoring methodology

A 5-point Likert-type scale that ranged from “never true” to “always true” was used. Most items were reverse scored, with “never true” score as 5 points and “always true” scored as 1 point. A few items were positively scored. The total score was the sum of all items, with higher points representing better cancer-related PF.

#### Expert consultation

After creating the potential items pool, expert consultation was conducted by sending an email to assess the accuracy and importance of the items and proposing modification suggestions. We selected 15 psychology experts from the ACT field; however, 12 experts ended up being involved in the consultation. There were five men and seven women, with ages ranging from 30 to 55 years (with an average of 43.1 ± 8.6 years). They had been involved in psychology for at least 5 years, and most are currently active in the ACT field. Regarding academic qualifications, one was an undergraduate, and the rest had a master’s or doctorate degree. Each expert evaluated the items independently.

In the first round of consultation, the experts recommended we add some items about self as context and being present, and split some items with double meanings, so that the number of items increased to 23 after this consultation. We then conducted the second round of expert consultation. After this consultation, we adjusted the items appropriately, modified the ambiguous items, adjusted the order of the items, and selected the most representative items. For example, “Even if I feel ill, I can still live a normal life” changed to “Even if I feel ill, I still try to live like before I got sick,” and “I experience a lot of pain when I think about or feel certain things because of my tumor” changed to “I feel pain for suffering from a tumor.” Meanwhile, according to experts’ suggestions, we put together items that expressed the same concept. Finally, the initial questionnaire with 23 items was generated after two rounds of consultations.

#### Pilot test

The pilot test took a sample of 15 inpatients from a tertiary cancer hospital, which was used to clarify ambiguous items, and delete items that were hard to understand or with multiple meanings. No incomprehension or ambiguity were discovered. As a result, a pilot questionnaire with 23 items was left.

### Procedure

Two master’s students from the research team distributed the survey face-to-face between November and December 2021. Before the survey, participants were informed about the purpose of this study, the requirements for participation, potential risks/benefits, and their right to terminate participation at any time. The researchers started the survey once informed consent was obtained. The survey was conducted anonymously, and participants participated in the survey free of charge.

### Data analysis

Data analysis was performed using the IBM SPSS software version 26.0 (IBM, Armonk, NY, United States). First, item-total correlations were used to test whether all items were consistent with the questionnaire. Inconsistent items were deleted based on the results. Second, the cases were divided into a high score group (the first 27%) and a low score group (the last 27%) according to the total score of the CPFQ, and then the scores of all items in the two groups were compared. Items with no significant differences indicating a lack of identification were deleted. Third, Kaiser-Meyer-Olkin and Bartlett’s test of sphericity was used to test whether the data were appropriate for factor analysis. Fourth, principal component analysis (PCA) was used to explore the latent structure of the CPFQ. The criteria for dimensions and item selection were as follows (Wu, 2010): (1) eigenvalues >1; (2) factors containing three or more items; (3) items load strongly (*>*0.40) onto factors; and (4) items do not cross-load onto two or more factors.

### Results and discussion

Based on the item analysis, the following four items were removed because the correlation coefficient (r) between the item and the total score was 0.233, 0.189, 0.254, and 0.280, respectively: Item 7: “The tumor made me realize what is important in life”; item 8: “We still live a happy life although we are in distress”; item 10: “I try not to think about the changes that cancer treatment may bring”; and item 16: “Even if I am ill, I still try to attend family, friends, or classmate gatherings.” Item analysis ranked the total scores of 231 patients from low to high and assigned the first 27% as the low score group and the last 27% as the high score group. The *t*-test of two independent samples was used to detect the differences between the 23 items in the high and low score groups. The results showed no significant difference on item 7 and item 8, which further indicated that the identification of these items was low and should be deleted.

In the Kaiser-Meyer-Olkin test, an *r-*value of 0.831 indicated that the data was suitable for factor analysis. A Bartlett test of sphericity (*χ*^2^ = 2879.375, df = 171, *p <* 0.001) indicated that the analysis model was appropriate. Therefore, it was acceptable to adopt factor analysis to test the construct reliability of this scale.

**Figure 1:**
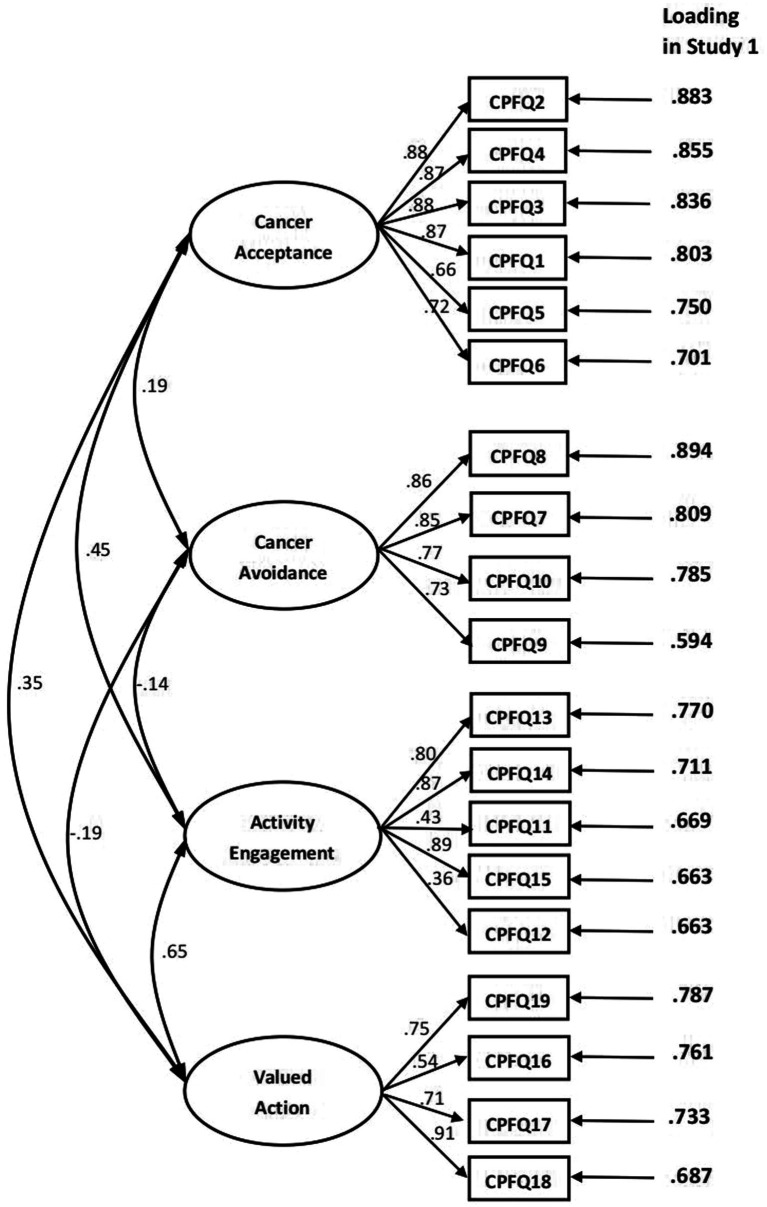
Confirmatory factor analysis in Study 2, and rotated(promax) factor loading in Study 1 with principal component analysis.

Applying PCA and varimax orthogonal rotation, we set parameters and extracted four factors with eigenvalues >1 and a cumulative variance interpretation rate of 68.939%. Four factors all contained at least four items, and the loading of each item was more than 0.59 (see Figure 1). According to the content of the items, the four factors were named as cancer acceptance (six items, *M* = 20.08, *SD* = 5.64, skewness = −0.262, kurtosis = −0.376), cancer avoidance (four items, *M* = 10.31, *SD* = 2.97, skewness = −0.048, kurtosis = −0.239), activity engagement (five items, *M* = 18.05, *SD* = 3.70, skewness = −0.059, kurtosis = −0.501), and valued action (four items, *M* = 16.01, *SD* = 2.63, skewness = −0.402, kurtosis =0.824). The items of the original Chinese form are shown in Appendix.

Following a series of data analyses, Study 1 resulted in a 19-item scale with four factors. This proposed model evaluated the PF of cancer patients, and its structure was inconsistent with the existing measuring tools for PF. For example, the CPAQ-8 contains two factors, that is pain willingness and activity engagement (Fish et al., 2010). The Personalized Psychological Flexibility Index includes three dimensions (avoidance, acceptance, and harnessing; Kashdan et al., 2020). This four-factor structure of the CPFQ was based on one sample; hence study 2 was conducted to validate the factor structure in another dataset.

## Study 2: Validation

To validate the four-factor structure and test the validity and reliability of the 19-item CPFQ, Study 2 collected another dataset. A CFA was conducted to test the four-factor model of PF. Furthermore, previous studies indicated a positive correlation between PF and life meaning but a negative relationship between anxiety and experiential avoidance (Lv et al., 2021). Therefore, meaning in life (assessed by the Meaning in Life Questionnaire) and death anxiety (assessed by the Templer’s Death Anxiety Scale) were used to evaluate the concurrent validity of the CPFQ, and experiential avoidance (assessed by the AAQ II) was used to evaluate the convergent validity of the CPFQ.

### Materials and methods

#### Participants

Inpatients were invited to participate in this survey from a tertiary cancer hospital in Hunan Province, China. The selection criteria of the participants were consistent with Study 1. A total of 285 questionnaires were sent to patients, and 252 patients completed the whole questionnaire, with a valid response rate of 88.4%. Of all the patients, 130 were men and were aged between 22 and 90 with an average age of 56.0 ± 11.3 years. Eighty six percent of patients (*n* = 217) were married, 5.2% (*n* = 13) were single, and 8.7% (*n* = 22) were divorced or widowed. As for educational background, 27.8% (*n* = 70) completed primary school or below, 40.1% (*n* = 101) junior high school, 17.5% (*n* = 44) senior high school, and 14.7% (*n* = 37) college and above. The cancer stages of the sample were as follows: 5.9% (*n* = 15) patients had stage I, 25% (*n* = 63) stage II, 42.5% (*n* = 107) stage III, 13.9% (*n* = 35) stage IV, and 12.7% (*n* = 32) were not reported.

To validate the model, the subjects-to-parameters ratio could not be lower than 5:1, and the total number of subjects needed to be over 200. The new sample (*n* = 252) in Study 2 reached a 5.7:1 subjects-to-parameters ratio, which was appropriate for testing a model with 44 parameters, consisting of 19 factor loadings, 19 error variances, and six factor correlations.

#### Procedure and measures

The survey was distributed face-to-face by three master’s students from the research team between January and March 2022. Before the survey, participants were informed about the purpose of this study, the requirements of participation, potential risks/benefits, and their right to terminate participation at any time. The researchers started the survey once informed consent was obtained. In order to evaluate concurrent validity and convergent validity, patients were required to complete the Meaning in Life Questionnaire, the Templer’s Death Anxiety Scale, and the AAQ II.

#### Meaning in life questionnaire

Meaning in life (MIL) was measured by the Meaning in Life Questionnaire (MLQ; Steger et al., 2006). The questionnaire contained the following two subscales: (1) The presence of meaning (MLQ-P), which assessed the extent to which meaning is experienced in a respondent’s life using statements such as “I understand my life’s meaning,” and (2) search for meaning (MLQ-S), which assessed a respondent’s desire to find and understand MIL using statements such as “I am searching for meaning in my life.” The original questionnaire had 10 items (five items for each of the two subscales) scored using a 7-point Likert scale ranging from one point (absolutely untrue) to seven points (absolutely true). Higher scores on the MLQ suggested that respondents were more likely to perceive and find MIL. Chinese scholars had previously translated and modified the questionnaire. The Chinese version, with five items for the MLQ-P and four items for the MLQ-S, was reported to have satisfactory reliability and validity (Liu and Gan, 2010). Finally, the MLQ was used to analyze the concurrent validity of the CPFQ.

#### Templer’s death anxiety scale

The Templer’s Death Anxiety Scale (T-DAS; Templer, 1970) assessed death anxiety and was used to analyze the concurrent validity of the CPFQ. The scale consisted of 15 items with dichotomous responses (true/false). Nine items were forward scored, and six were reverse scored, and higher scores indicated greater death anxiety. This scale was reported to have test–retest reliability of 0.83 and reasonable internal consistency of 0.76. This scale had been translated into multiple languages and used in several countries. The Chinese version of T-DAS contained four factors. These were labeled (1) Stress and pain, (2) Emotion, (3) Cognitive, and (4) Awareness of Time Passing. The translated measure demonstrated good reliability and validity with an estimated internal consistency of Cronbach’s *α* = 0.71 (Yang et al., 2012).

#### Acceptance and action questionnaire II

The Acceptance and Action Questionnaire II (AAQ-II; Bond et al., 2011) was a general measure of experiential avoidance and was used to analyze convergent validity with CPFQ. AAQ-II was developed by Bond et al. in 2011, a unidimensional scale with seven items based on the seven points Likert scale, ranging from one (never true) to seven (always true). The total score was summed over the seven items, with higher scores representing greater experiential avoidance and lower PF. AAQ-II had good test–retest reliability and good internal consistency (α = 0.88). The Chinese version of AAQ-II was modified by Cao et al. in 2013 (Cao et al., 2013), which had established a good content validity index, and acceptable internal consistency with Cronbach’s *α* = 0.88.

### Data analysis

Data analysis was performed using Amos version 23.0, SPSS version 26.0, and M*plus* version 8.3. The construct validity was identified by the confirmatory factor analysis (CFA), and the criteria for indexes that were used to assess the goodness of fit of the model as follows: 1 < *χ*^2^/*df* < 3, comparative fit index (CFI) > 0.90, goodness-of-fit index (GFI) > 0.90, and root-mean-square error of approximation (RMSEA) < 0.08 (Wu, 2010). The equivalence of the model across gender was examined by invariance testing, and the criterion for indices that were used to evaluate the adequacy of the fit of the model as follows: ΔCFI was <0.01, ΔRMSEA was <0.015 (Cheung and Rensvold, 2002).

Descriptive statistical analysis was used to examine the mean, standard variation, skewness, and kurtosis of the four factors. In order to assess the concurrent validity of the questionnaire, Pearson’s *r* between CPFQ, MIL, and T-DAF was calculated. In order to assess the convergent validity of the questionnaire, Pearson’s *r* between CPFQ and AAQ-II was calculated. Internal consistency of the CPFQ was examined using Cronbach’s alpha coefficient. Finally, split-half reliability *R* was evaluated by the correlation coefficient *r* between the odd and even items (R=2r/1+r).

### Results and discussion

#### Construct validity analysis

To obtain the construct validity of the four-factor structure developed from Study 1, CFA with the maximum likelihood method was conducted by Amos 23.0. The results showed a good fit to the data of Study 2, *χ*^2^ = 297.572, *χ*^2^/*df* = 2.343, *p* < 0.001, CFI = 0.948, GFI = 0.900, RMSEA = 0.073, 90% CI = 0.062–0.084. Configural or factorial invariance analysis was conducted by M*plus* 8.3 across gender group to determine whether the factor structure of the CPFQ was the same for both men and women. The results revealed the model fit the data reasonably well, with ΔCFI <0.01, and ΔRMSEA <0.015 (Table 1). Therefore, formal and measurement invariance across gender was evidenced for the CPFQ.

**Table 1:** Invariance testing across gender group.

Invariance level	*χ* ^2^	df	*χ*^2^/df	CFI	RMSEA	ΔCFI	ΔRMSEA
Configural	442.154	284	1.557	0.876	0.066		
Metric	466.107	299	1.559	0.869	0.067	−0.007	0.001
Scalar	491.209	314	1.564	0.861	0.067	−0.008	−0.001

The standardized coefficients of each path are shown in Figure 1. Descriptive analysis revealed that the distributions were relatively normal for the overall CPFQ (*M* = 63.75, SD = 10.65, skewness = 0.112, kurtosis = −0.016) and its four factors: cancer acceptance (*M* = 19.80, SD = 5.73, skewness = −0.320, kurtosis = −0.187), cancer avoidance (*M* = 10.15, SD = 3.31, skewness = 0.290, kurtosis = 0.110), activity engagement (*M* = 17.70, SD = 3.67, skewness = 0.029, kurtosis = −0.430), and valued action (*M* = 16.10, SD = 2.63, skewness = −0.352, kurtosis = 0.656).

#### Concurrent and convergent validity analysis

Pearson’s correlational analysis was conducted to explore the association between the CPFQ and other measures. The overall CPFQ was significantly positively associated with the presence of meaning (*r* = 0.519), search for meaning (*r* = 0.257), and MIL (*r* = 0.456), and negatively associated with death anxiety (*rs* = −0.449 to −0.591), and experiential avoidance (*r* = −0.704), which provided evidence that the overall CPFQ was measuring the essence of psychological flexibility and could estimate mental and behavioral health of cancer patients. Table 2 shows that most correlation coefficients were significant, ranging from −0.159 to.747, except for Cancer Avoidance. Cancer Avoidance was weakly correlated with AAQ-II (*r* = −0.175, *p* < 0.01) and not correlated with other measures.

**Table 2:** Means, standardized deviation (SD) of measures, and correlations between overall of CPFQ and its four subscales, two dimensions of MLQ, four dimensions of T-DAS, and AAQ-II.

	MLQ-P	MLQ-S	MLQ	DAS Stress and pain	T-DAS Emotion	T-DAS Cognitive	T-DAS Awareness of time passing	T-DAS	AAQ-II
Mean	24.62	19.10	43.71	2.83	2.08	1.67	1.03	7.60	25.56
SD	4.681	4.227	7.707	1.673	1.718	1.041	0.820	4.332	9.641
*r* with									
Cancer Acceptance	0.397**	0.084	0.287**	−0.538**	−0.513**	−0.574**	−0.465**	−0.637**	−0.747**
Cancer Avoidance	0.116	0.012	0.077	−0.067	−0.056	−0.039	−0.010	−0.059	−0.175**
Activity Engagement	0.496**	0.323**	0.478**	−0.405**	−0.291**	−0.395**	−0.409**	−0.444**	−0.561**
Valued Action	0.399**	0.392**	0.457**	−0.333**	−0.254**	−0.159*	−0.219**	−0.309**	−0.219**
Overall CPFQ	0.519**	0.257**	0.456**	−0.532**	−0.457**	−0.496**	−0.449**	−0.591**	−0.704**

**p < 0.01, *p < 0.05.

#### Reliability analysis

The Cronbach’s *α* coefficient of the whole CPFQ was 0.885, and the Cronbach’s *α* coefficient for Cancer Acceptance, Cancer Avoidance, Activity Engagement, and Valued Action was 0.927, 0.874, 0.823, and 0.849, respectively, which indicated that the items were internally consistent. The odd and even items were summed and the correlation coefficient was statistically significant with *r* = 0.898, and split-half reliability was 0.946.

#### Effects of gender, age, and cancer stage on CPFQ

Table 3 shows the effects of gender, age, and cancer stage on the overall CPFQ and its four dimensions. Independent *t*-tests showed a significant difference in Valued Action (*t* = −2.590, *p* < 0.05) and no significant difference in the overall CPFQ and the other three dimensions between men and women. In terms of cancer stage, ANOVA displayed statistical differences in the overall CPFQ and its three dimensions (*F* > 5.789, *p* < 0.005), except for Cancer Avoidance. Table 3 shows that age was related to Activity Engagement, Valued Action, and the overall CPFQ (*F* > 2.784, *p* < 0.018).

**Table 3:** Effects of gender, age, and cancer stage on the overall and four dimensions of CPFQ.

Variables	*n*	Cancer Acceptance	Cancer Avoidance	Activity Engagement	Valued Action	Overall CPFQ
Gender						
Male	130	19.35 (5.36)	10.26 (3.34)	17.63 (3.80)	15.69 (2.57)	62.94 (10.59)
Female	122	20.27 (6.08)	10.03 (3.30)	17.77 (3.55)	16.54 (2.63)	64.61 (10.68)
*t*		−1.266	0.547	−0.301	−2.590	−1.250
Value of *p*		0.207	0.585	0.764	0.010	0.212
Age (years)						
20–30	3	16.33 (7.37)	7.67 (4.04)	19.00 (5.29)	18.00 (3.46)	61.00 (12.29)
31–40	29	19.24 (6.58)	10.93 (3.47)	19.00 (3.55)	16.69 (2.58)	65.86 (12.13)
41–50	42	20.33 (6.36)	10.05 (3.87)	18.69 (4.13)	17.12 (2.87)	66.19 (11.49)
51–60	94	20.66 (4.62)	10.47 (3.58)	17.27 (3.38)	15.78 (2.52)	64.17 (9.32)
61–70	65	19.52 (6.14)	9.74 (2.51)	17.92 (3.42)	15.82 (2.49)	63.00 (10.41)
>70	19	16.68 (5.45)	9.42 (2.43)	14.68 (3.16)	15.26 (2.45)	56.05 (10.61)
*F*		1.963	1.231	4.590	2.784	2.894
Value of *p*		0.085	0.295	0.001	0.018	0.015
Cancer stage						
I	15	24.07 (4.27)	10.27 (3.67)	22.13 (3.11)	19.40 (1.40)	75.87 (7.92)
II	63	20.30 (4.48)	10.48 (3.64)	18.51 (2.84)	15.83 (2.57)	65.11 (9.39)
III	107	19.65 (5.56)	9.98 (2.94)	17.39 (3.67)	16.01 (2.39)	63.04 (9.80)
IV	35	17.20 (7.23)	9.80 (3.28)	16.11 (3.80)	15.57 (2.51)	58.69 (12.78)
*F*		5.789	0.438	12.126	10.257	10.664
Value of *p*		0.001	0.726	<0.001	<0.001	<0.001

Overall, the 19-item CPFQ with four dimensions showed a good model fit in a second Chinese sample. The results demonstrated good reliability, construct validity, concurrent validity, and convergent validity of the CPFQ. The overall CPFQ and its dimensions were positively correlated with MIL, and negatively correlated with death anxiety and experiential avoidance. However, Cancer Avoidance showed non-significant correlations with MIL and death anxiety. This outcome may be due to cancer patients’ characteristics, whose attitudes toward cancer change over time. Future studies are needed to continue to validate the four-factor structure of the CPFQ in a bigger sample.

This study suggested women showed higher Valued Action. In China, most women undertake more roles and responsibilities than men, as they take care of many people. Even if they do not accept cancer, avoid cancer issues, and avoid socializing, they still do something based on their values, such as taking the doctor’s advice to live longer.

It is worth noting that age was correlated with the CPFQ, especially for activity engagement and valued action. The younger the patient was, the more willing they were to participate in activities and do something worthwhile. However, in terms of the overall CPFQ, patients aged 31–50 show higher PF. The possible reason is that middle-aged patients are mentally more mature than younger patients and more responsible than older people. Therefore, considering the small sample size of some subgroups, future studies are necessary to explore this interesting phenomenon further.

Furthermore, the current study also revealed that patients with advanced cancer had lower PF than those with early cancer. It may be because early cancer is easier to treat, has a better prognosis, and is easier to recovery. Patients acquire posttraumatic growth after this life event and tend to cherish work, life, families, and friends more. It is interesting that irrespective of the cancer stage, they all had an attitude of avoiding cancer. One possible explanation for this phenomenon is that according to Chinese traditional culture, Chinese people are extremely sensitive about their illness and consider cancer a disgrace.

In this study, we recruited patients with different types of cancer, but we did not analyze the impact of cancer types on PF. Because there were many types of cancer and the sample size was not relatively small, cancer was difficult to classify by types. If the classification is very specific, the sample size of each category will be very small, and if classified by tumor region, the severity of diseases in the same category will vary greatly, e.g., head and neck cancer includes oral and thyroid cancer; however, the severity of these diseases is completely different. Although the extent of the impact of different types of cancer on PF is unknown, it clearly has an impact. Because different types of cancer have different symptoms and prognoses, symptoms and prognoses will affect PF.

## General discussion

The current study describes the development and preliminary validation of the CPFQ, an instrument to measure PF in cancer patients. Initial scale development resulted in a 23-item instrument, which was reduced to 19 items based on item-scale correlations and PCA. After item analysis and PCA, a four-factor structure of the CPFQ indicated four dimensions of psychological flexibility of cancer patients: Cancer Acceptance, Cancer Avoidance, Activity Engagement, and Valued Action. The PCA revealed a four-dimension questionnaire consistent with the concept of PF (Cherry et al., 2021). Confirmatory factor analysis indicated a good model fit on the four-factor structure; in other words, the construct validity was satisfactory. Concurrent validity was expressed as correlations between CPFQ, MIL, and death anxiety (T-DAS) were moderate. Convergent validity, as these constructs were supposed to share some common features, expressed as the correlation between CPFQ and AAQ-II was acceptable. The internal consistency and split-half reliability were beyond the specified standard. The results showed that the CPFQ had a clear factor structure and good psychometric properties in Chinese samples. Therefore, the questionnaire is valuable and beneficial for research on the PF of cancer patients.

The CPFQ reflects both attitudes and behaviors toward cancer. The four dimensions of the CPFQ represented PF in terms of cancer acceptance, cancer avoidance, social contact, and behavior orientation. The ability to live a valuable life despite a cancer diagnosis is a type of PF related to cancer. Different from other life events, cancer is a life-threatening disease, and individuals’ responses should be different from other stress events. Therefore, the PF of cancer patients may have its own essence and characteristics. Examining the four specific dimensions of the CPFQ, the former two dimensions mainly assessed the patients’ attitudes toward cancer, and the latter two dimensions mainly measured their behavioral tendencies after a cancer diagnosis. These contents reflected not only the nature of PF, such as acceptance, cognitive fusion, being present, values, and action, but also the characteristics of cancer patients.

Differing from other measures of psychological flexibility, the CPFQ measures (1) individuals’ psychological response to cancer and their attitudes toward the psychological response; (2) one’s emotional and behavioral tendencies when thinking of cancer treatment; and (3) individuals’ social interaction and behavior change after cancer. Compared with other questionnaires for measuring PF, the CPFQ has similarities and differences. For example, the CPAQ includes two dimensions, namely pain willingness (feeling little need to avoid or control painful experiences) and activity engagement (the degree to which one engages in life’s activities regardless of pain; McCracken et al., 2004; Fish et al., 2010). The similarities are that both questionnaires measure patients’ attitudes and behaviors toward diseases (cancer vs. chronic pain). The difference is that the content of CPFQ is more comprehensive, including not only psychological responses and behavioral orientation to diseases, but also avoidance reactions and valued actions to diseases.

The CPFQ contributes significantly by providing a valuable tool that measures components of psychological flexibility and verifies psychological flexibility from cancer patients’ perspectives. As described in the “Materials and Methods” section, the items of the CPFQ were generated from both the literature review and the theoretical definition of psychological flexibility. We also refer to some items from other measurement instruments of psychological flexibility, such as the CPAQ (Fish et al., 2010) and MPFI (Rolffs et al., 2018). Moreover, we interviewed cancer patients about their feelings, attitudes, and behavior change after a cancer diagnosis. Hence, we support that the CPFQ is a questionnaire with a solid theoretical foundation and comprehensive content.

In summary, a new measurement instrument of PF was developed and validated in two samples. To our knowledge, this is the first cancer-specific psychological flexibility measurement that includes attitude and behavior components, which could provide a more accurate assessment of PF among cancer patients and help health care providers develop personalized and targeted interventions in the PF field. The CPFQ was a reliable and valid tool to evaluate the PF of cancer patients with a four-factor structure: Cancer Acceptance, Cancer Avoidance, Activity Engagement, and Valued Action. Moreover, this questionnaire has a good readability and a reasonable length with 19 items. We believe that the CPFQ can be used as a valuable measurement in the psychological flexibility field.

## Limitations and future directions

The present study forms a preliminary version of the CPFQ. However, there are still some limitations. First, there may be sampling bias. The samples of Study 1 and Study 2 were from the same hospital, and it would be important to verify the reliability and validity of the current questionnaire among different groups. Future studies could apply this questionnaire to other groups, such as cancer patients from a general hospital, to validate our results. Second, one dimension of the questionnaire, namely Cancer Avoidance, showed unsatisfactory validity values, which needs further exploration in future research. Finally, our study did not evaluate test–retest reliability, because most inpatients had been discharged at the time of the retest, and there may exist a deviation between the online questionnaire survey and the face-to-face survey. When applying the questionnaire in the future, a small sample (such as 30) of cancer patients could be selected for the re-test reliability test.

## Conclusion

The CPFQ includes four dimensions: Cancer Acceptance, Cancer Avoidance, Activity Engagement, and Values Actions, and it was proven to be a reliable and valid measure of psychological flexibility in cancer patients.

## Data availability statement

The raw data supporting the conclusions of this article will be made available by the authors, without undue reservation.

## Ethics statement

Ethical review and approval was not required for the study on human participants in accordance with the local legislation and institutional requirements. The patients/participants provided their written informed consent to participate in this study.

## Author contributions

M-jO, X-hX, and SS contributed to the original idea and concepts. HC and F-rC completed data collection and analysis. M-jO wrote the first draft. X-hX and SS revised the manuscript. All authors approved the final version of manuscript for submission.

## Funding

This work was supported by grants from Hunan Provincial Natural Science Foundation of China (grant No. 2021JJ40327 and 2018JJ6110).

## Conflict of interest

The authors declare that the research was conducted in the absence of any commercial or financial relationships that could be construed as a potential conflict of interest.

## Publisher’s note

All claims expressed in this article are solely those of the authors and do not necessarily represent those of their affiliated organizations, or those of the publisher, the editors and the reviewers. Any product that may be evaluated in this article, or claim that may be made by its manufacturer, is not guaranteed or endorsed by the publisher.

## Appendix

Items of the original Chinese form of the Cancer-related Psychological Flexibility Questionnaire.

**Table:**

癌症接纳	1.因为得了肿瘤，我觉得自己是一个不幸的人
	2.我为患有肿瘤感到沮丧
	3.我为患有肿瘤感到羞愧
	4.我为患有肿瘤感到痛苦
	5.做任何事情前，我总会优先考虑到我的疾病
	6.我无法忍受疾病或治疗带来的改变
癌症回避	7.我尽量不去想肿瘤给我带来的糟糕感觉
	8.我会尽量避免可能导致疼痛等不适的因素
	9.我会尽量避免肿瘤治疗可能导致的副反应
	10.我会尽力去控制或摆脱因肿瘤带来的痛苦感觉和想法
活动参与	11.我不去面对周围的人
	12.我不去跟别人谈论我的疾病
	13.即便患病，我仍然尽量参加热爱的活动或事业
	14.即便患病，我仍然保持与自己喜欢的人来往
	15即便身体不适，我仍然尽量像患病前一样生活
基于价值的行动	16.我积极配合医生的治疗
	17.我采取了更加健康的生活方式
	18.我尽可能承担自己的角色
	19.我努力完成自己的心愿
